# Differential Regulation of Microglial Activation in Response to Different Degree of Ischemia

**DOI:** 10.3389/fimmu.2022.792638

**Published:** 2022-01-28

**Authors:** Hao Gao, Furong Ju, Rujuan Ti, Yue Zhang, Shengxiang Zhang

**Affiliations:** Gansu Key Laboratory of Biomonitoring and Bioremediation for Environmental Pollution, School of Life Sciences, Lanzhou University, Lanzhou, China

**Keywords:** transcriptome, microglia, activation, proliferation, ischemia, inflammation

## Abstract

Microglia are primary immune cells within the brain and are rapidly activated after cerebral ischemia. The degree of microglial activation is closely associated with the severity of ischemia. However, it remains largely unclear how microglial activation is differentially regulated in response to a different degree of ischemia. In this study, we used a bilateral common carotid artery ligation (BCAL) model and induced different degrees of ischemia by varying the duration of ligation to investigate the microglial response in CX3CR1^GFP/+^ mice. Confocal microscopy, immunofluorescence staining, RNA sequencing, and qRT-PCR were used to evaluate the de-ramification, proliferation, and differential gene expression associated with microglial activation. Our results showed that 30 min of ischemia induced rapid de-ramification of microglia but did not have significant influence on the microglial density. In contrast, 60 min of ischemia led to a significant decrease in microglial density and more pronounced de-ramification of microglial processes. Importantly, 30 min of ischemia did not induce proliferation of microglia, but 60 min of ischemia led to a marked increase in the density of proliferative microglia. Further analysis utilized transcriptome sequencing showed that microglial activation is differentially regulated in response to different degrees of ischemia. A total of 1,097 genes were differentially regulated after 60 min of ischemia, but only 68 genes were differentially regulated after 30 min of ischemia. Pathway enrichment analysis showed that apoptosis, cell mitosis, immune receptor activity and inflammatory-related pathways were highly regulated after 60 min of ischemia compared to 30 min of ischemia. Multiple microglia-related genes such as *Cxcl10*, *Tlr7*, *Cd86*, *Tnfrsf1a*, *Nfkbia*, *Tgfb1*, *Ccl2* and *Il-6*, were upregulated with prolonged ischemia. Pharmacological inhibition of CSF1 receptor demonstrated that CSF1R signaling pathway contributed to microglial proliferation. Together, these results suggest that the proliferation of microglia is gated by the duration of ischemia and microglia were differentially activated in responding to different degrees of ischemia.

## Introduction

Ischemic stroke is one of the main causes of death and disability worldwide. Acute interruption of the local blood flow leads to energy failure and a series of pathological events, including excitotoxicity, oxidative/nitrative stress, disruption of blood brain barrier (BBB), and activation of microglia (1–4). Among these events, activation of microglia plays a key role in the pathological progression of ischemic damage. For the past decades, the roles of microglial activation in ischemic conditions has been well explored and elucidated (5–7). However, there are controversies regarding microglial functions in ischemic brain. Several studies indicate that activated microglia are neuroprotective under ischemic conditions (8, 9; wang et al., 2018), but other evidences suggest that reactive microglia can aggravate ischemic injury and compromise functional recovery in ischemic stroke (10–13). Our recent study has also shown that activated microglia is detrimental to the recovery of ischemic tissue (14). Therefore, microglia can be beneficial or detrimental to ischemic tissue depending on ischemic conditions.

The exact function of microglia may be related to their activation state. As resident immune cells within the central nervous system system (CNS), microglia exhibit different degree of activation and show diverse functions under physiological or pathological conditions. In a healthy brain, microglia surveil the microenvironment in the brain parenchyma, participate in the formation and pruning of neuronal synapses, and cross-talk with other glial cells to maintain CNS homeostasis and modulate neuronal functions (15–18). After being activated, microglia undergo series of morphological and functional changes, including de-ramification, proliferation, releasing pro- or anti-inflammatory factors, and enhancing phagocytosis (19–27). In addition, activated microglia can also release matrix metalloproteinases, superoxide and other neurotoxic elements. All these factors may contribute to the protective or deleterious roles of microglia upon activation.

Previous studies have shown that the degree of microglial activation differed between different ischemic conditions (11, 28). Consistently, our study indicate that microglial activation can last for at least one week in focal cerebral ischemia (29), but are activated only in earlier stage in transient ischemia (30). Nevertheless, how microglial activation is differentially regulated in response to a different degree of ischemia remains largely unrevealed.

In this study, we investigated the regulatory mechanisms of microglial activation in a mouse model of global ischemia by taking advantage of CX3CR1^GFP/+^ mice and transcriptome sequencing (RNA-sequencing). Our results revealed that the proliferation of microglia is gated by the duration of ischemia, and microglial activation is differentially regulated in response to different degrees of ischemia.

## Materials and Methods

### Animals

Transgenic mice Thy1-YFP line H (expressing yellow fluorescent proteins in layer 5 pyramidal neurons, JAX #003782), CX3CR1^GFP/+^ (expressing green fluorescent proteins in microglia) and C57/BL6 mice were purchased from the Jackson Laboratory. The animals were bred in the Laboratory Centre for Basic Medical Sciences, Lanzhou University. Mice had free access to food and clean water and were housed in an environment of regular 12-h light/12-h dark cycle at 22 ± 2°C. Mice of both sexes at the age of 3 months and weighing 20-30 g were used in this research. Throughout the study, all experiments were implemented strictly following the institutional animal guidelines of Lanzhou University, China.

### Global Cerebral Ischemia Model

We used bilateral common carotid artery ligation (BCAL) to induce global ischemia as described in previous studies (30–33). The degree of ischemia was achieved by controlling the duration of ligation (30 min or 60 min). Briefly, mixed solution of ketamine (150 mg/kg body weight) and xylazine (25 mg/kg xylazine body weight) was intraperitoneally injected to deeply anesthetize the mice, and then a thinned-skull cranial window above somatosensory cortex was made by using a high-speed dental drill. Animals were gently laid in supine position, and an incision was made over the cervical region. The common carotid arteries were carefully exposed and encircled with surgical sutures. To induce global ischemia, the surgical sutures were tightened for 30 or 60 minutes, and then the sutures were loosened to perform reperfusion. The animals used in this study were divided into three groups: Sham group, 30 min BCAL group, and 60 min BCAL group.

### Administration of GW2580

The GW2580 (LC Laboratories) was used as an inhibitor to inhibit the CSF1R cascade as described by previous studies (34, 35). GW2580 was suspended in 0.5% hydroxypropyl methylcellulose and 0.1% Tween 80. The solution was dosed orally to mouse (at the body weight of 75 mg/kg) after ischemia at a daily interval for 3 days.

### Immunofluorescence Staining

To label newly proliferative cells during global ischemia-reperfusion, bromodeoxyuridine (BrdU, 50mg/kg; Sigma) was intraperitoneally injected to the mice two times per day, started from the 12^th^ hour after BCAL. 2 days after BCAL, the mice brain tissues were extracted, fixed with 4% paraformaldehyde solution and sectioned.

BrdU immunofluorescence staining was performed to label the proliferative cells after ischemia as described before (29). Briefly, brains sections (30 μm) were rinsed in PBS for 10 min and treated with 2M HCl at 37°C for 30 min, neutralized with 0.1 M borate buffer (pH 8.5) for 3×15 min, and then incubated overnight with a rat monoclonal antibody against BrdU (1:500; AbD Serotec). After washing three times, the brain sections were stained using rhodamine-conjugated goat anti-rat second antibody (1:100; ZSGB-BIO) for 1 h at room temperature. The sample was then sealed with PBS/glycerol mixture (2: 3) and examined under a confocal microscope. Microglia (green) overlapped with BrdU-positive cells (red) have been distinguished as proliferative microglia.

### Two-Photon Imaging and Confocal Imaging

Intravital two-photon imaging was used to measure blood velocity during ischemia and after reperfusion. After being subjected to deep anesthetization, the skull of the animal was exposed, tightly glued to a custom-made metal frame and then fixed to a steel plate. A 2×2 mm^2^ area of which center at -1.5 mm from bregma and 2.0 mm from midline was positioned on the skull and thinned to ~25 μm by using a high-speed dental drill. During the surgery, body temperature of the mouse was maintained at 37 ± 0.5°C by a heating pad. Real-time two-photon microscopy was performed by using an Olympus FV1000 microscope (Olympus, Japan). The wavelength of the exciting laser was adjusted to 890 nm and the Z step size was set to 0.75 μm. Images were acquired at zoom 2 through water-immersion objective lens (×25/1.05; Olympus). For blood flow velocity (reflected by the velocity of red blood cells) measurement, 20 μl Texas Red-dextran (10 mg/ml, Invitrogen, USA) was injected intravenously to the mouse *via* tail vein, then an image was taken by repeated line scanning after drawing a straight line along the center of a vessel (10~15 µm in diameter). The ImageJ software (http://rsb.info.nih.gov/ij/) was used to measure the velocity of blood flow. The blood velocity of all the animals from three groups (Sham, 30 min BCAL, 60 min BCAL) was measured before the induction of global ischemia and 3 h after reperfusion.

For cell density analysis, confocal images of 30-60 μm sections were acquired by using an Olympus confocal microscope (FV1000). Stack projection was made by using a Z project function in ImageJ. The red (BrdU^+^) and green (GFP^+^) channel were merged with the Merge Channels function. ROIs were chosen in the somatosensory cortex (2.5-4 mm from the midline). 10 ROIs from each mouse were used to calculate the density of microglia. The number of microglia was quantified with the Cell Counter function of ImageJ software, and the microglial density was then calculated.

### Skeletonization Analysis of Microglia

Brain tissue slices of 60 μm ranging from 1 mm to 2 mm of posterior bregma point were collected. Every fifth slice was chosen and microglia from 8 areas per slice were quantified. Skeletonization of GFP^+^ microglia was performed using a plugin of ImageJ software as described before (30). Briefly, the images were processed by using a plugin (“Plugin” > “Process” > “Smooth 3D”; radius, 0.3-0.8). The total number of microglial process endpoints and total length of microglial processes of single cell were quantified by using an analytic skeleton plugin (http://imagejdocu.tudor.lu), and skeletonized images were generated by a Skeletonization plugin (2D/3D).

### Real-Time Fluorescence Quantitative PCR

For qRT-PCR analysis, CX3CR1^GFP/+^ mice were first transcardially perfused with PBS, and then fresh cortical tissues from all three groups (n = 5/group) were obtained under a dissecting microscope. RNA Extraction Kit (TaKaRa) and Nanodrop (Thermo Scientific) were used to extract and quantitate RNA, respectively. The agarose gel (2%) electrophoresis was used to check the integrity of RNAs, and RNAs were reversely transcribed by Reverse Transcription Kit with gDNA Eraser (Applied Biosystems). Real-time fluorescence quantitative PCR was performed by using iTaq™ Universal SYBR^®^ Green supermix (Bio-Rad) to analyze the expression level of genes. The custom-designed gene-specific primers (GENEWIZ) are shown as follows: *Ccl2* (FW, TTAAAAACCTGGATCGGAACCAA, RV, TCCACCACCCTGTTGCTGTA), *Cxcl10* (FW, CTGCCGTCATTTTCTGCCTC, RV, TTCAAGCTTCCCTATGGCCC), *Tlr7* (FW, TCTGCGTGTCAAGGGGTATGTC, RV, GTGCCAAGGTCAAGAACTT

CCAG), *Cd86* (FW, CAGAACTTACGGAAGCACCCA, RV, ATAAGCTTGCGTCTCCACGG), *Tnfrsf1a* (FW, CGATAAAGCCACACCCACAA, RV, ACCTTTGCCCACTTTTCACC), *Il-6* (FW, TAGTCCTTCCTACCCCAATTTCC, RV, TTGGTCCTTAGCCACTCCTTC), *Nfkbia* (FW, TAGTCCTTCCTACCCCAATTTCC, RV, TTGGTCCTTAGCCACTCCTTC), *Tgf-β* (FW, GGCGATACCTCAGCAACCG, RV, CTAAGGCGAAAGCCCTCAAT), *Arg1* (FW, TCACCTGAGCTTTGATGTCG, RV, CTGAAAGGAGCCCTGTCTTG), *CD206* (FW, CAAGGAAGGTTGGCATTTGT, RV, CCTTTCAGTCCTTTGCAAGC), *Cx3cl1* (FW, GTGCTGACCCGAAGGAGAAA, RV, CACCCGCTTCTCAAACTTGC), *Il4* (FW, CAAACGTCCTCACAGCAACG, RV, AGGCATCGAAAAGCCCGA), *Ccr3* (FW, CCAGCTGTCAGCAGAGTAAA, RV, CTCACCAACAAAGGCGTAGA), *Csf1r* (FW, GCAGTACCACCATCCACTTGTA, RV, GTGAGACACTGTCCTTCAGTGC), *Csf1* (FW, AGTATTGCCAAGGAGGTGTCAG, RV, ATCTGGCATGAAGTCTCCATTT), *Il34* (FW, CTTTGGGAAACGAGAATTTGGAGA, RV, GCAATCCTGTAGTTGATGGGGAAG), *Pu.1* (FW, CAGAAGGGCAACCGCAAGAA, RV, GCCGCTGAACTGGTAGGTGA), *Cebpa* (FW, AGCTTACAACAGGCCAGGTTTC, RV, CGGCTGGCGACATACAGTAC), *Runx1* (FW, CAGGCAGGACGAATCACACT, RV, CTCGTGCTGGCATCTCTCAT) and *GAPDH* (FW, CGTGCCGCCTGGAGAAACCTG, RV, AGAGTGGGAGTTGCTGTTGAAGTCG). The glyceraldehyde-3-phosphate dehydrogenase (*GAPDH*) was chosen as the reference gene. Relative quantity of mRNA levels was evaluated by the process of 2-^ΔΔCT^.

### RNA Sequencing

Considering the regional heterogeneity of microglial cells (36), only the cortical tissues of CX3CR1^GFP/+^ mice were extracted after transcardiac perfusion. For RNA sequencing, the total RNA was extracted using TRIzol^®^ reagent (Invitrogen, Carlsbad, CA, USA). RNA degradation and contamination were monitored on 1% agarose gel to check the purity using a Nano Photometer^®^ and spectrophotometer (IMPLEN, CA, USA). The concentration was measured with the Qubit^®^ RNA Assay Kit in a Qubit^®^ 2.0 Fluorometer (Life Technologies, CA, USA). RNA integrity was assessed with the RNA Nano 6000 Assay Kit of the Agilent Bioanalyzer 2100 system (Life Technologies, CA, USA). Then, RNA-Seq was performed on the mRNA isolated from samples of sham-control tissues and ischemic tissues. Three biological replicates were used for each group. Using NEBNext^®^ Ultra™ RNA Library Prep Kit for Illumina^®^ (NEB, USA), libraries were generated and sequenced according to the manufacturer’s recommendations. Briefly, mRNA was isolated from a total of 3 μg RNA using magnetic poly-T oligo beads. The AMPure XP system (Beckman Coulter, Beverly, USA) was used to obtain the cDNA fragment 150~200 bp from the library fragments. The PCR products were purified using the AMPure XP system, and the quality of the library was assessed using Agilent Bioanalyzer 2100 system. Clean reads were obtained by removing reads containing adapter and poly-N, as well as other low quality reads from the raw data. Reference genome and genes model annotation files were downloaded from the genome website. HTSeq (v 0.6.1) was used to count the read numbers mapped to each gene. Quantification was achieved by digital gene expression (DGE) analysis, and DGE-Seq reads were assembled into transcripts. Genes’ abundance was estimated in Fragments Per Kilobase of transcript per Million mapped reads of each gene (FPKM) using the method described by Trapnell et al. (37). Annotations of biological pathways and gene ontology are respectively referred from the Kyoto Encyclopedia of Genes and Genomes database (KEGG, https://www.genome.jp/kegg/), and the Gene Ontology project (http://www.geneontology.org). RNA-seq data are available at Sequence Read Archive (SRA) accession PRJNA777759, or they can be accessed by the following link: https://apac01.safelinks.protection.outlook.com/?url=https%3A%2F%2Fwww.ncbi.nlm.nih.gov%2Fsra%2FPRJNA777759&data=04%7C01%7C%7Cc60a5e72072c49909bd608d9a1c6f539%7C84df9e7fe9f640afb435aaaaaaaaaaaa%7C1%7C0%7C637718698154505889%7CUnknown%7CTWFpbGZsb3d8eyJWIjoiMC4wLjAwMDAiLCJQIjoiV2luMzIiLCJBTiI6Ik1haWwiLCJXVCI6Mn0%3D%7C1000&sdata=pRGIh63J0dwqGUJpz81EYwRQx0odi7npehf1XUsc58A%3D&reserved=0.

### Data Analysis

Statistical analysis was performed using GraphPad Prism9.0 software. For data with a small sample size (n = 4~6 for each group), two-tailed unpaired t-test were used for comparison between two groups; one-way ANOVA was performed for comparison among three or more groups. For data with a sample size of 40 or 50, data were first tested for normality using the D’Agostino-Pearson omnibus normality test. If the data showed a normal distribution and homogeneity of variances could not be rejected, one-way ANOVA was performed for comparison among three or more groups; if not, nonparametric test (one-way ANOVA with the Kruskal-Wallis test for three or more groups) were used. Results were presented as mean ± standard error of the mean, *p<0.05 and **p<0.01. The bioinformatic analysis of RNA-seq data was performed with packages in R environment (https://www.r-project.org/). Analysis of differentially expressed genes and gene enrichment analysis were performed respectively by DESeq2 (38) and clusterProfiler package (39). Biological and statistical significance were screened together by adjusted p-value (padj < 0.05), FPKM (Fragments Per Kilobase of transcript per Million mapped reads; FPKM > 1) and the value of Fold Change of genes (Fold Change > 1 or Fold Change < 0.5).

## Results

### Neuronal Damage Induced by Different Degree of Ischemia

To verify the effect of bilateral common carotid artery ligation on cerebral blood flow, we measured the velocity of cortical blood flow in CX3CR1^GFP/+^ mouse during surgery using two-photon *in vivo* imaging (**Supplementary Figure 1A**). Results showed that during the ligation surgery, the blood flow velocity dropped significantly to a low level compared to that of pre-surgery in both 30 min and 60 min BCAL group (**Supplementary Figures 1B, C** **;** for 30 min BCAL group: 1278.1 ± 84.5, 22.3 ± 5.2, 1282.8 ± 67.8, 1200.1 ± 105.7 μm/s for Pre, BCAL, 3 h and 2 days after BCAL, respectively; for 60 min BCAL group: 1328.2 ± 110.8, 22.8 ± 4.1, 1305.6 ± 66, 1193.9 ± 93.3 μm/s for Pre, BCAL, 3 h and 2 days after BCAL, respectively; n = 5 mice for each group; **p<0.01). At both 3 h and 2 days after BCAL, the blood flow was restored to a level of the pre-surgery (**Supplementary Figures 1B, C**). Such results indicate that the BCAL surgery can induce ischemic stroke efficiently.

To explore the outcomes of different degrees of ischemia, we performed two-photon *in vivo* imaging in Thy1-YFP line H transgenic mice. Results showed that both 30 min and 60 min of ischemia induced notable beaded-like damage to apical dendrites (**Supplementary Figure 2A**). Moreover, these structural damages were partly restored after two days of BCAL. We then performed Fluoro-Jade C staining in C57/BL6 mince to distinguish degenerating neurons. Results showed that compared to Sham group, both 30 min and 60 min BCAL caused degeneration of cortical neurons (**Supplementary Figure 2B**). However, the density of degenerating neurons in 60 min BCAL group was significantly higher than that of 30 min BCAL group (**Supplementary Figure 2C**; 22.5 ± 9.1/mm^2^ for 30 min BCAL group, and 290.3 ± 27.3/mm^2^ for 60 min BCAL group; n = 4 mice for each group; *p<0.05). Together, these facts indicated that the distinct degree of global ischemia can be confirmed by different degree of neuronal damage.

### Activation of Microglia Is Differentially Regulated by the Degree of Ischemia

**Figure 1:**
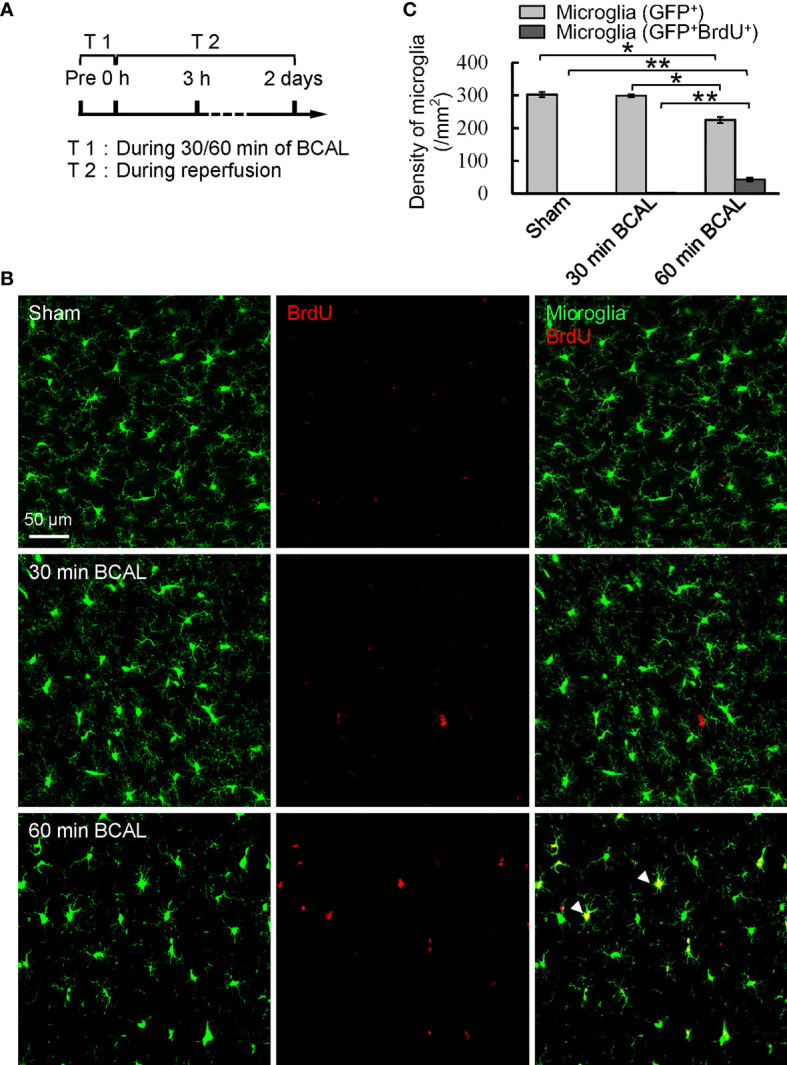
Effect of different degree of ischemia on microglial density and proliferation. **(A)** Timeline showing the time points for ischemia, reperfusion, and measuring microglial density and proliferation. **(B)** Confocal images show microglia (green) and BrdU labeled cells (red) in Sham, 30 min BCAL, and 60 min BCAL group. Arrowheads indicate GFP^+^/BrdU^+^ cells that were identified as proliferative microglia. **(C)** Statistical plot showing the densities of GFP^+^ and GFP^+^/BrdU^+^ cells in Sham, 30 min BCAL and 60 min BCAL group (n = 6 for each group, *p<0.05, **p<0.01).

To evaluate the impact of different degree of ischemia on microglial activation, we quantified the density of cortical microglia at 3 h and 2 days after BCAL **(** **Figure 1A** **;** **Supplementary Figure 3A** **)**. Results showed that the density of cortical microglia at 3 h after BCAL showed no significant difference between Sham, 30 min BCAL and 60 min BCAL groups (**Supplementary Figures 3B, C** **;** 300.5 ± 5.9/mm^2^ for Sham group, 292.1 ± 2.7/mm^2^ for 30 min BCAL group, and 278.6 ± 7.9/mm^2^ for 60 min BCAL group; n = 6 mice for each group), suggesting that microglial density was largely unchanged within 3 h following ischemia. Data of 2 days after BCAL showed that the microglial density of the mice in the 30 min BCAL group showed no significant difference to that of the Sham group **(** **Figures 1B, C** **;** 302.3 ± 7.4/mm^2^ for Sham group versus 298.5 ± 4.7/mm^2^ for 30 min BCAL, n = 6 mice for each group**)**. However, the microglial density of the 60 min BCAL group was significantly decreased compared to both 30 min BCAL and Sham group **(** **Figures 1B, C** **;** 225.1 ± 9.5/mm^2^ for 60 min BCAL; n = 6 mice for each group; *p<0.05, **p<0.01**)**. To investigate the proliferation of microglia after BCAL, we used BrdU staining to label proliferative microglia. Results showed that there is no significant difference in the density of BrdU^+^ microglia between the 30 min BCAL group and Sham group **(** **Figure 1C** **;** 2.3 ± 0.2/mm^2^ for 30 min BCAL versus 1.0 ± 0.3/mm^2^ for Sham**)**. In contrast, a significant increase in proliferative microglia was observed in the 60 min BCAL group compared to both 30 min BCAL and Sham group **(** **Figure 1C** **;** BCAL 60 min 43.2 ± 5.1/mm^2^ **p<0.01**)**. These results suggest prolonged but not transient ischemia induces a reduction in microglial density and promotes microglial proliferation at 2 days after reperfusion.

**Figure 2:**
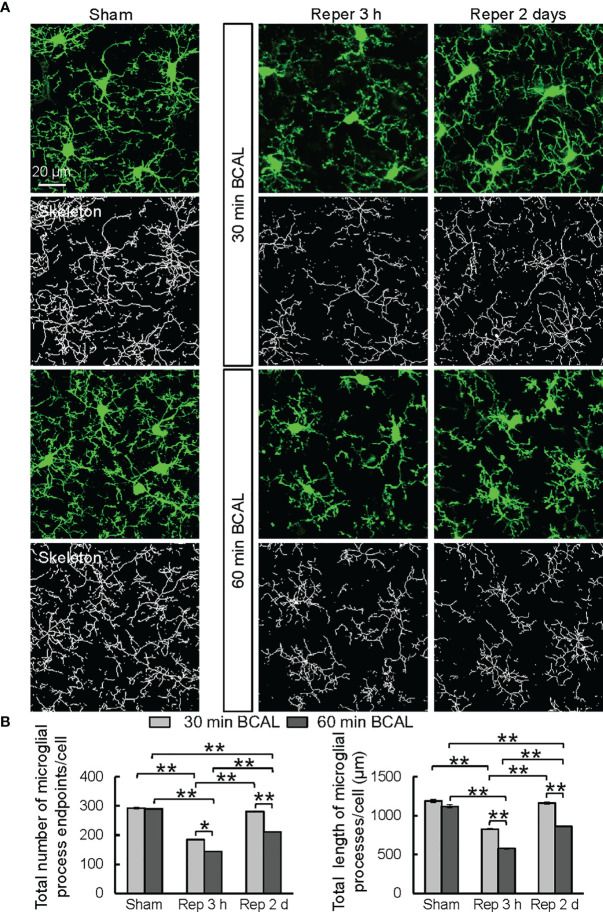
Microglial morphology is differentially regulated by the degree of ischemia. **(A)** Representative confocal images showing morphologies of microglia (green) and skeletonized microglia in Sham group, and at 3 h, 2 days after ischemia in 30 min BCAL group and 60 min BCAL group. **(B)** Quantification of total number of microglial process endpoints and total length of microglial processes per cell. Note that both 30 and 60 minutes of ischemia have decreased number of microglial process endpoints and length of microglial processes. There is no significant difference in the total number of microglial process endpoints and the total length of microglial processes of each cell between 30 min BCAL group and Sham group, but there are significant reductions in 60 min BCAL group compared to Sham group. (n = 50 microglia from 5 mice for each group; *p<0.05, **p<0.01).

To investigate the impact of different degrees of ischemia on the morphology of microglia, we analyzed the microglial morphology using a skeleton analysis plugin of ImageJ on confocal microscopy images (**Figure 2A**). Results showed that at 3 h after reperfusion the total number of microglial process endpoints and the total length of microglial processes of each cell were significantly decreased in both 30 min BCAL group and 60 min BCAL group compared to Sham group. Notably, the total number of microglial process endpoints and the total length of microglial processes of each cell in 60 min BCAL were significantly lower than those of the 30 min BCAL **(** **Figure 2B** **;** total number of microglial process endpoints of each cell: 184.1 ± 1.2 for 30 min BCAL vs 143.5 ± 1.1 for 60 min BCAL; the total length of microglial processes of each cell: 826.0 ± 6.2 μm for 30 min BCAL vs 578.2 ± 6.7 μm for 60 min BCAL; *p<0.05, **p<0.01). We further analyzed microglial morphology at 2 days after BCAL. We found that there were significant differences between 30 min BCAL and 60 min BCAL in both the total number of microglial process endpoints and the total length of microglial processes of each cell. However, when we compared these data to those of the Sham group, we found that there is no significant difference in the total number of microglial process endpoints and the total length of microglial processes of each cell between 30 min BCAL group and Sham group, but there are still significant reductions in 60 min BCAL group when compared to Sham group **(** **Figure 2B** **;** total number of microglial process endpoints of each cell: 280.9 ± 1.4 for 30 min BCAL vs 211.1 ± 1.5 for 60 min BCAL; the total length of microglial processes of each cell: 1161.0 ± 15.8 μm for 30 min BCAL vs 860.4 ± 7.0 μm for 60 min BCAL; **p<0.01**)**. These data indicate that 30 min of ischemia led to a significant de-ramification of microglia at 3 h after reperfusion, but the microglial morphology was restored at 2 days after ischemia. In contrast, 60 min BCAL induced a more pronounced microglial de-ramification, and microglial morphology did not restore at 2 days after ischemia. Together, these data suggested that the complexity of microglial morphology is differentially regulated by the degree of ischemia.

Apart from the morphological changes, activated microglia also undergo phenotypic changes in different ischemia conditions (11, 24, 40–42). To explore the effect of different degrees of ischemia on the microglial polarization, we performed immunofluorescent staining to distinguish M1 (CD16/32 positive) and M2 (CD206 positive) microglia. Results showed that the density of CD16/32 positive microglia was significantly higher in the 60 min BCAL group than that in the 30 min BCAL group (**Supplementary Figures 4A, C**; 39.5 ± 3.7/mm^2^ for 30 min BCAL group vs 93.3 ± 11.4/mm^2^ for 60 min BCAL group, n = 4 mice for each group. **p<0.01). Similarly, CD206 positive microglia also showed a higher density in the 60 min BCAL group compared with 30 min BCAL group (**Supplementary Figures 4B, C**; 5 ± 0.7/mm^2^ for 30 min BCAL group vs 14.8 ± 1.4/mm^2^ for 60 min BCAL group, n = 4 mice for each group. **p<0.01). These results indicated that the density of both M1 and M2 microglia was increased in higher degree of ischemia. In addition, the density of CD16/32 positive microglia was higher than that of CD206 positive microglia in both the 30 and 60 min BCAL groups, suggesting that pro-inflammatory response of activated microglia was more pronounced than anti-inflammatory response at 2 days after ischemia.

### Gene Expressions Were Differentially Regulated by Different Degree of Ischemia

**Figure 3:**
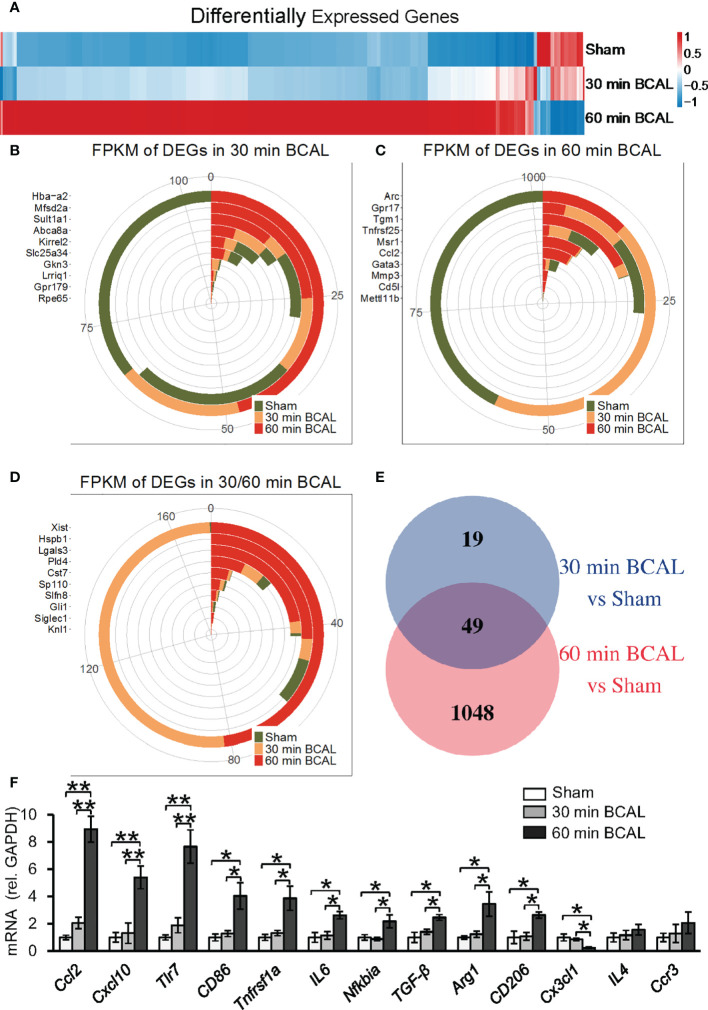
Differentially expressed genes after ischemic stroke. **(A)** Heatmap showing the gene expression between different groups. Genes with similar expression pattern were clustered. Colors show the relative up- and downregulation of each gene. **(B–D)** Top 10 genes with the largest Fold Change in 30 min BCAL group, 60 min BCAL group, and both groups. The expression of the gene is presented as Fragments Per Kilobase of transcript per Million mapped reads (FPKM). **(E)** Venn diagram showing the number of DEGs in each group. The overlapping region indicates the number of genes that were expressed in both 30 min BCAL and 60 min BCAL group. **(F)** The validation of DEGs using qRT-PCR. The mRNA expression level of each gene was normalized to *GAPDH* (n = 4 mice for each group; *p<0.05, **p<0.01). Multiple genes that related to microglial activation were differentially expressed in 60 min BCAL. These data are consistent with DEG data.

**Figure 4:**
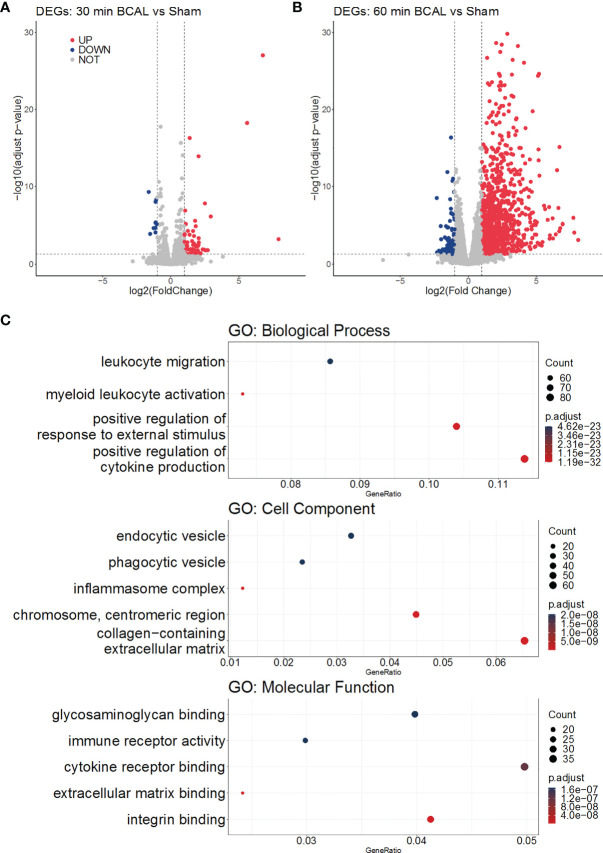
Gene Ontology analysis of DEGs. **(A, B)** Volcano plots show the upregulation and downregulation of genes in the 30 min and 60 min BCAL groups, respectively. **(C)** GO enrichment of DEGs in 60 min BCAL. The top 5 terms of each GO aspect are displayed in order as: Biological Process, Cell Component, Molecular Function. The color indicates the significance of the term, and the size of the ball indicates the Gene Ratio in the term.

Above data indicate that both the proliferation and morphology of microglia are regulated by the degree of ischemia. To explore the molecular mechanism, we used RNA sequencing to evaluate the effect of different degrees of ischemia on gene expressions on the transcriptomic level. Differentially expressed genes (DEGs) were filtered to distinguish the expression profiles in mice of ischemic groups from that of the Sham group (**Figure 3** **;** n = 3 for each group). In total, 68 genes in the 30 min BCAL group and 1,097 genes in the 60 min BCAL group were differentially expressed compared to the Sham group, respectively (**Figure 3E**). Among them, a total of 59 genes were significantly upregulated and 9 genes were downregulated in the 30 min BCAL group; in comparison, 1,008 genes were significantly upregulated, and 89 genes were downregulated in the 60 min BCAL group **(** **Figures 4A, B** **)**. These results suggest that different levels of ischemia have significant effects on gene expression patterns of cortical tissue, and the number of induced genes is greater than the number of suppressed genes. To infer the biological processes that might occur in different degrees of ischemia by investigating the functions of DEGs, we then examined the 10 genes with the largest variation (Fold Change) in expression levels (top 10 DEGs) at different degree of ischemia **(** **Figures 3B–D** **)**. Data showed that *Xist*, *Cst7*, *Sp110* that related to microglial function were found both in 30- and 60 min BCAL **(** **Figure 3D** **)**, and *Gpr17*, *Tgm1*, *Msr1* that related to microglial activation were exclusively found in 60 min BCAL **(** **Figure 3C** **)**. However, no microglial activation-related genes were found in the top 10 exclusive DEGs of the 30 min BCAL group, indicating that among the DEGs with the largest Fold Change, there were more genes associated with microglial activation in the 60 min BCAL group.

Further, we examined the expression levels of a series of genes associated with microglial activation. The result of differential expression analysis showed that genes including *Ccl2*, *Cxcl10*, *Tlr7*, *Cd86*, *Tnfrsf1a* and *Arg1* were significantly upregulated in 60 min BCAL but not 30 min BCAL. The DEG data were then validated using qRT-PCR **(** **Figure 3F** **)**. These results suggest that the expression of regulatory genes of microglial activation was significantly upregulated by a higher degree of ischemia. These data are consistent with imaging data showing enhanced de-ramification and proliferation of microglia following prolonged ischemia.

In addition, we compared the genes expression profile of 60 min BCAL group to that of the 30 min BCAL group. Results showed that 733 genes were differentially expressed in 60 min BCAL group. Among them, 81 genes were downregulated and 652 genes were up-regulated (**Supplementary Figure 5A**). We further explored the expression of several microglial marker genes, and the results showed that factors including *Ccl2*, *Cxcl10*, *Cd86, Cd68, Csf2rb, Pu.1, Cd68, and Arg1* were significantly upregulated in 60 min BCAL group (**Supplementary Figure 5B**). Changed expressions of these genes indicated that the microglial activation was significantly enhanced in the prolonged ischemia. Interestingly, the expressions of anti-inflammatory factors (*Tgfb1, Arg1*) and pro-inflammatory factors (*Cxcl10, Tnf*) were both elevated in 60 min BCAL group. Together with the results of immunofluorescence staining (**Supplementary Figure 4**), these results implied that the number of both pro- and anti-inflammatory cortical microglia were increased in the 60 min BCAL group. In addition, considering that the integrity of the BBB can affect microglial activation, we investigated the function of the BBB related cells at the transcriptional level. The expression of endothelial cell markers and pericyte markers were then examined (43). The results showed that genes including *Ly6a*, *Angptl4*, *Flnc*, *Dcn*, and *Emp1* et al. were differentially expressed in the 60 min BCAL group. Such results implies that prolonged ischemia may lead to functional changes of BBB.

### Over-Representation Analysis of DEGs in Different Degree of Ischemia

**Figure 5:**
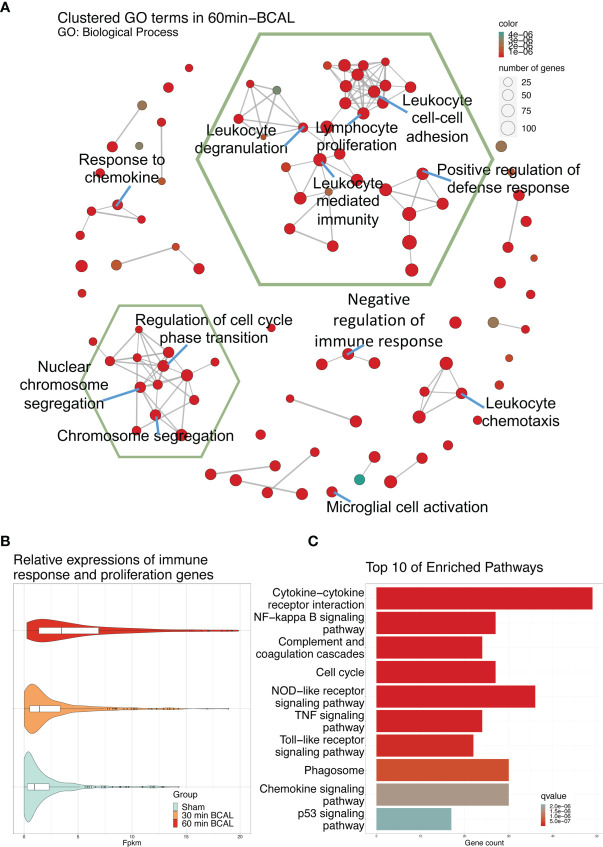
Effect of 60 min of ischemia on the activation of biological processes and signaling pathways. **(A)** Correlation analysis of the top 100 significantly enriched GO: Biological Process terms. Terms which share ancestor terms are linked and clustered. The green hexagonal box circles the two largest clusters. The color of the node indicates the significance of the term, and the size of the node indicates the number of genes in the term. **(B)** Expression of DEGs in the two largest clusters of GO: Biological Process terms in 60 min BCAL. **(C)** Top 10 most significant enriched terms after KEGG enrichment of DEGs in 60 min BCAL. Color indicates the significance of the term.

To explore the biological processes in which these differentially expressed genes may be involved, we took advantage of the gene ontology database. GO database provides annotations that map genomic products to three aspects: biological process (BP), cellular component (CC), and molecular function (MF). We mapped DEGs of 30- and 60 min BCAL group to the GO database. The data showed that DEGs of 30 min BCAL did not enrich any specific GO term. However, DEGs of 60 min BCAL were mapped to the GO database and results showed that several terms of both BP, CC, and MF were significantly enriched. Terms including positive regulation of cytokine production, myeloid leukocyte activation, positive regulation of external stimuli, cytokine receptor binding, immune receptor activity, chromosome centromeric region, inflammasome complex, phagocytic vesicle are classic terms that characterize activation and/or reaction of immune cells, cell mitosis, phagocytic and tissue inflammation (**Figure 4C**). To dig deeper into the relevance between microglial activation and immune response exhibited in 60 min BCAL, we conducted an association analysis of the BP terms (44). The result show that the top 100 enriched BP terms were clustered into 2 main functional modules: cell mitosis and immune and/or inflammatory response (**Figure 5A**). Importantly, the expressions of genes that enriched in the 2 main functional modules were significantly increased in 60 min BCAL (**Figure 5B**). These data suggested that immune response and proliferation genes were specifically enhanced following prolonged ischemia.

**Figure 6:**
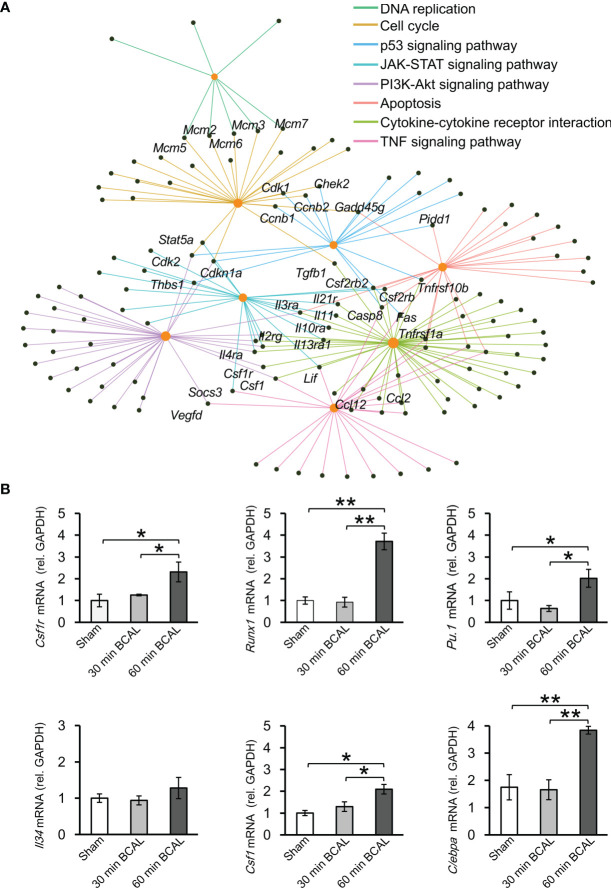
Regulatory network and key regulatory factors in 60 min ischemia. **(A)** Regulatory network of KEGG terms in 60 min BCAL. Orange nodes indicate KEGG terms, and black nodes indicate overlapping genes. **(B)** qRT-PCR was used to validate the result of RNA sequencing. The expression level of mRNA was normalized to *GAPDH*. *CSF1* related genes including *CSF1R*, *Runx1*, *PU.1* and *Cebpa* were upregulated in 60 min ischemia, and the expression level of *IL34* showed no significant difference between each group. These data are in agreement with DEG data. (n = 4 mice for each group; *p < 0.05 and **p < 0.01).

To associate DEGs with specific functional pathways, we utilized KEGG database to performed functional enrichment and trace genes functions by mapping them to signaling pathway annotations. Results show that no specific KEGG term was enriched by DEGs in 30 min BCAL group. In contrast, for 60 min BCAL, cascades that regulate chemotaxis, immunity, cellular survival/death, inflammation, phagocytosis, and cell mitosis including Cytokine-cytokine receptor interaction, NF-kappa B signaling, Phagosome, Apoptosis, p53 signaling pathway and Cell cycle were among the top 10 enriched pathways (**Figure 5C**). We further performed functional enrichment analysis of genes associated with immune response and cell proliferation and compared the results of the analysis with those of the overall DEGs in 60 min BCAL group. The result showed that pathways such as TNF signaling pathway, PI3K-Akt signaling pathway, JAK-STAT signaling pathway, Apoptosis, p53 signaling pathway, Cell cycle, and DNA replication formed a regulatory network (**Figure 6A**). Colony-stimulating factor1 (CSF1) and its receptor CSF1R are key genes controlling proliferation, differentiation and activation of microglia (45, 46). Importantly, these two genes were nodal genes in this regulatory network, and both are shared by TNF signaling and PI3K-Akt signaling pathway (**Figure 6A**).

KEGG database indicates that CSF1 is a downstream molecule of TNF signaling pathway and an upstream molecule of PI3K-Akt signaling pathway (**Supplementary Figures 6A, B** **)**. These two signaling pathways are in the upstream position of Cell cycle signaling pathway, and their interaction plays an important regulatory role for cell proliferation (47, 48). To evaluate the expression of genes related to CSF1 signaling, we quantified the expression of *Csf1r*, *Runx1*, *Pu.1*, *Csf1*, *Cebpa*, and *Il34*. Results showed that *Csf1r*, *Runx1*, *Pu.1*, *Csf1*, *Cebpa* were upregulated in 60 min BCAL group compared to those in 30 min BCAL group and Sham group (**Figure 6B**), but the expression of *Il34* did not show significant differences among the three groups. These results of qRT-PCR are in agreement with those of DEG analysis.

**Figure 7:**
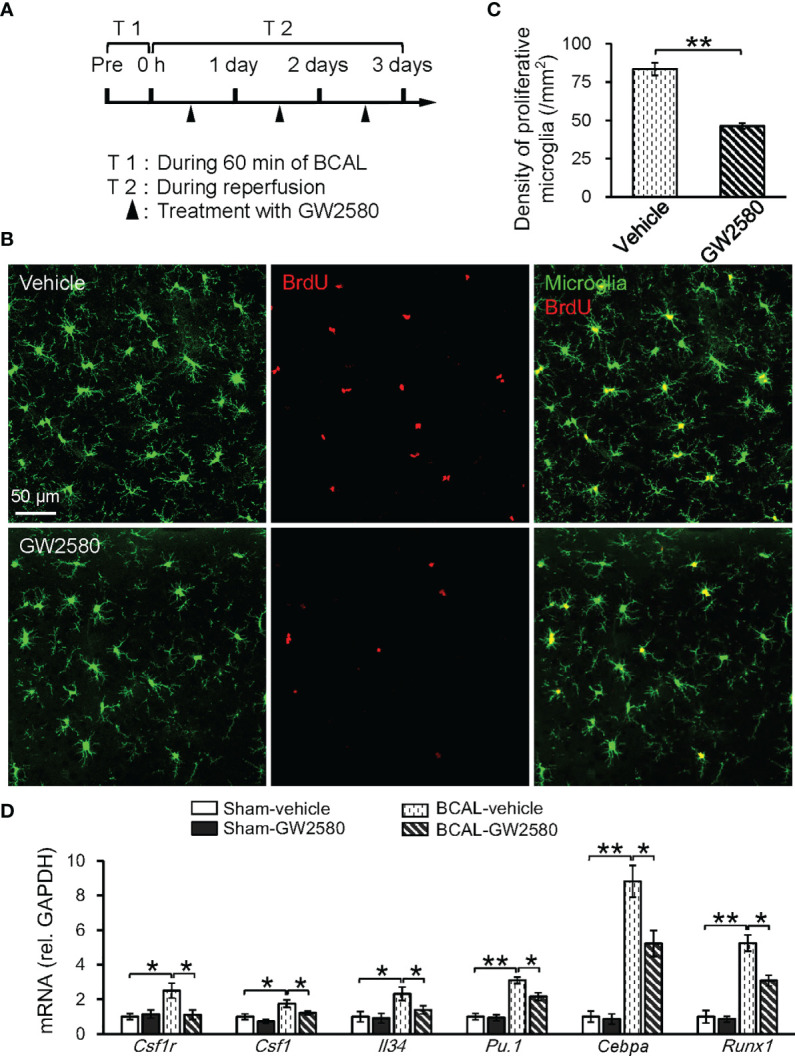
Proliferation of microglia induced by 60 min ischemia was suppressed by CSF1R inhibitor GW2580. **(A)** Timeline showing ischemia, reperfusion and GW2580 treatment. Filled arrows show the timepoints of intragastric administration of GW2580. **(B)** Confocal images showing microglia (green) and BrdU labeled proliferative cells (red) in Vehicle and GW2580 group. **(C)** Quantitative data showing the densities of proliferative microglia in Vehicle and GW2580 groups. Note that GW2580 treatment significantly inhibited microglial proliferation induced by 60 min BCAL (n = 6 mice for each group; **p<0.01). **(D)** qRT-PCR showing the effect of GW2580 treatment on the mRNA levels of CSF1-related genes. Note that the expressions of these genes showed no difference with or without GW2580 in Sham groups but were suppressed by GW2580 in ischemic groups (n = 4 mice for each group; *p<0.05, **p<0.01).

We further investigated whether the CSF1 signaling played a role in enhancing the microglial activation following 60-minute BCAL by using GW2580 to inhibit CSF1R signaling (**Figure 7A**). The result of BrdU immunofluorescent staining showed that the density of proliferative microglia in the BCAL-GW2580 group was significantly lower than that of the BCAL-Vehicle group (**Figures 7B, C** **;** density: 46.3 ± 1.8/mm^2^ for BCAL-GW2580 versus 83.5 ± 4.2/mm^2^ for BCAL-Vehicle; n = 6 mice for each group; **p<0.01). Consistently, the expressions of CSF1R signaling-related genes (*Csf1r*, *Runx1*, *Pu.1*, *Csf1*, *Cebpa*, *and Il34*) were significantly down-regulated (**Figure 7D**). These results suggested that the CSF1R signaling pathway played a key role in the proliferation of microglia induced by prolonged ischemia.

## Discussion

Microglia are the main resident immune cells within central nervous system. Activated microglia play a critical role in the pathological process of ischemic stroke (11, 24). One of the characteristic properties is that microglia can undergo morphological changes after activation, as manifested by the de-ramification of their highly dynamic processes. In this study, we evaluated the effects of different degrees of global ischemia (30 min BCAL vs 60 *min* BCAL) on microglia morphology in cerebral cortices. The results showed that both 30 min and 60 min of global brain ischemia triggered a significant de-ramification of microglia at 3 h after ischemia. Interestingly, the morphology of microglia in the 30 min BCAL group showed no significant difference from that of the Sham group after 2 days of BCAL, but the morphology of microglia in the 60 min BCAL group remained de-ramified at 2 days after BCAL. These data are consistent with our earlier report that microglial morphology can recover after transient ischemia (30). In addition, our data are in agreement with previous studies showing that ischemia-induced de-ramification of microglia can be restored after a period of reperfusion and the activation level of microglia is directly reflected by microglial morphology (22, 49).

Researchers have classified the activated microglia into distinct subtypes based on their functional differences. For example, phenotypes that express pro- or anti-inflammatory functions are respectively defined as classic (M1, pro-inflammatory) and alternative (M2, anti-inflammatory) phenotypes (26). Studies have revealed that the dynamic equilibrium between the microglial M1/M2 phenotype can have substantial effect on the outcomes of ischemic stroke (40, 50, 51). The polarization of microglia is regulated by a variety of factors and differentially polarized microglia show distinct functions (42, 52, 53). In our study, both transient and prolonged ischemia caused microglial activation and polarization. Moreover, the number of M1 microglia was higher than M2 microglia in both groups. This result suggested that polarized microglia were predominantly pro-inflammatory cells at 2 days after BCAL. These pro-inflammatory microglia can further in turn activate other microglia by secreting pro-inflammatory factors including IL-6, TNF-α, CD86, etc. Therefore, a higher degree of ischemia can enhance the polarization of M1 microglia and promote the microglial activation.

In addition to internal regulatory factors, microglial activation can also be modulated by neurons. For instance, CD200 expressed on the membrane of healthy neurons is reported to be necessary in maintaining the “resting” state of microglia through interacting with CD200 receptors located on microglial membrane (54–56). In our study, both 30 min and 60 min of BCAL can induce neuronal damage. However, at 2 days after ischemia, the density of degenerating neurons was notably greater in the 60 min BCAL group than that in the 30 min BCAL group. This result implied that the inhibitory effect of neurons on microglial activation was attenuated in 60 min BCAL compared to 30 min BCAL group. Thus, enhanced activation of microglia can be observed in a higher degree of ischemia.

In a healthy brain, the number of microglia is maintained by the co-working of apoptosis and proliferation and remains relatively stable, and a reduction in microglial density is usually coupled with the proliferation of microglia (57, 58). Under pathological conditions, microglia can enhance proliferative activity (20, 22, 29, 59). Here, we showed that transient global ischemia did not have a significant effect on microglial density and proliferative activity. However, prolonged ischemia resulted in a significant decrease in the density of microglia and an increase in proliferative microglia. Interestingly, the lower density of cortical microglia in 60 min BCAL suggested that although microglia have shown enhanced proliferative activity, the number of newborn microglia was not enough to counteract that of the decreased (apoptotic or necrotic) microglia. Together, these data suggest that proliferation of microglia is gated by the degree of ischemia, and enhanced proliferation of microglia only occurred in prolonged, but not in transient ischemia. Another consequence of reduced microglial density is the invasion of peripheral immune cells. Microglia can prevent the entry of immune cells into the brain parenchyma, and the reduction of microglia results in the aggregation of immune cells (34, 60). Studies have shown that the invading peripheral immune cells can enhance the neuroinflammatory response (61), and whether the activation state of microglia can be promoted in this process needs further investigation in future study.

We used RNA sequencing to explore the regulatory mechanisms involved in the activation of microglia. Consistent with other studies and our previous work (62–65), our results showed that the expression of genes was altered in both transient and prolonged ischemia. However, there is a remarkable difference in the number of DEGs (1,097 genes for 60 min ischemia vs 68 genes for 30 min ischemia). Our analysis indicates that DEGs of 30 min BCAL did not enrich any specific GO term or KEGG term. In contrast, DEGs of 60 min BCAL were enriched to multiple key terms that are associated with activation and/or reaction of immune cells, cell mitosis and tissue inflammation. Furthermore, KEGG enrichment analysis suggests that DEGs of 60 min BCAL are involved in key signaling pathways, such as TNF signaling pathway, JAK-STAT signaling, and Apoptosis. These results suggest that the gene expression profile in ischemic brain tissue was altered by ischemia, and this expression profile was differentially regulated by the degree of ischemia.

GO enrichment analysis shows that the top 100 enriched BP terms were clustered into 2 main functional modules: cell mitosis and immune and/or inflammatory response. Combining with the imaging data of microglial activation in 60 min BCAL, our results indicated an enhanced microglial activation and immune and/or inflammatory response with prolonged ischemia at both structural and transcriptomic levels. Previous studies indicate that signaling cascades such as TNF signaling, PI3K-Akt signaling and JAK-STAT signaling are deeply involved in neuroinflammation (66–68). Considering that the inflammatory response can promote microglial activation, the enhanced microglial activation in 60 min BCAL may be due to an exacerbated neuroinflammation (61, 69). Furthermore, a series of microglial activation-related genes were upregulated in prolonged ischemia. Several key regulators of microglial activation, including *Xist*, *Cst7*, and *Sp110* (70–72) were differentially upregulated in both 30- and 60 min BCAL group. Furthermore, signature genes of microglia, including *Cxcl10*, *Tlr7*, *Cd86*, *Tnfrsf1a*, *Nfkbia*, *Tgfb1*, *Iba1* (9, 43, 73–75), were significantly induced following prolonged ischemia. In addition, genes that related to microglial activation including *Ccl2*, *Il6*, *Arg1*, *Cx3cl1*, were also significantly upregulated in the 60 min BCAL group. Together, these results suggested that microglial activation was differentially regulated by the degree of ischemia, and an exacerbated inflammatory response in prolonged ischemia was associated with a higher degree of microglial activation.

As a secreted cytokine, CSF1 participates in the formation of a crucial cytokine network during inflammation, regulates both proliferation and differentiation of myeloid cells, and is necessary for microglial viability (34, 45, 46). Here we showed that *Csf1* and *Csf1r* were upregulated and were nodal genes in the regulatory network. Consistently, the expression levels of CSF1R signaling-related genes (*Runx1*, *Pu.1*, *Cebpa*) were also upregulated by 60 min of global ischemia. These genes are involved in regulating microglial activation and proliferation after ischemic injury (76–78). Using GW2580 as a pharmacological inhibitor of CSF1R, we validated that microglial proliferation following ischemia was CSF1 receptor-dependent. Together, these findings indicate that CSF1R and its related genes play a critical role in differential regulation of microglial activation following ischemia.

Another important regulator of microglial activation is the blood brain barrier 79. Our previous study showed that after 30 min BCAL, the interrupted BBB was gradually restored with reperfusion, and that the increased BBB permeability can enhance the activation of microglia (30). In our present study, we looked into the expressions of several genes that related to BBB integrity and function. We found that endothelial markers (*Angptl4*), and pericyte marker (*Cyp1b1*, *Emp1*, *S1pr3*, *Serping1*, and *Cxcl1*) were upregulated in prolonged ischemia. These genes have been reported to be highly associated with the breakdown and restoration of the BBB (32, 80–83). Therefore, the differential expression of these genes in the 60 min BCAL group suggested that the permeability of the BBB may not be restored at 2 days after BCAL, and subsequently promoted microglial activation. In future study, this issue should be further addressed on the histological level.

Another factor that is highly correlated with BBB integrity is the infiltration of peripheral macrophage/monocyte. Macrophage/monocyte infiltration may contribute to the activation of microglia and gene expression profile of ischemic tissue. The study here is based on whole cortical tissues and does not allow for the distinction between microglia and monocytes genes. Future studies using gene expression analysis on sorted isolated cells are warranted to characterize specific microglia or monocytes genes.

In summary, the present study suggests that the proliferation of microglia was gated by the duration of ischemia. Microglial activation is differentially regulated in response to different degrees of ischemia. Microglia-related genes were markedly induced by prolonged ischemia, and CSF1R-related signaling pathways play a key role in microglial proliferation. The findings in this study may provide insights into regulating microglial activation in clinical treatments of ischemic stroke.

## Data Availability Statement

The datasets presented in this study can be found in online repositories. The names of the repository/repositories and accession number(s) can be found below: https://www.ncbi.nlm.nih.gov/bioproject, PRJNA777759.

## Ethics Statement

The animal study was reviewed and approved by Ethics Committee of Lanzhou University.

## Author Contributions

HG, FJ, and SZ designed the research plan. HG, FJ, and YZ performed the experiments. HG, FJ, RT, and YZ analyzed the data. HG, FJ, RT, and SZ wrote the paper. All authors contributed to the article and approved the submitted version.

## Funding

This work was supported by grants from the National Natural Science Foundation of China (No. 81771324).

## Conflict of Interest

The authors declare that the research was conducted in the absence of any commercial or financial relationships that could be construed as a potential conflict of interest.

## Publisher’s Note

All claims expressed in this article are solely those of the authors and do not necessarily represent those of their affiliated organizations, or those of the publisher, the editors and the reviewers. Any product that may be evaluated in this article, or claim that may be made by its manufacturer, is not guaranteed or endorsed by the publisher.

## Supplementary Material

Supplementary Figure 1 Velocity of blood flow. **(A–C)** Cerebral blood flow before, during and after BCAL were shown as the velocity of red blood cells, respectively. Two-photon images show microglia (green) and vessels (red). Arrows indicate the vessels used for the measurement of blood flow velocity (n = 40 vessels from 5 mice for each group; a vs b, *p*<0.01).

Supplementary Figure 2 Neuronal damage after different degree of ischemia. **(A)** Beaded-like structural damage of neuronal dendrites induced by 30 min and 60 min BCAL. **(B)** Magnified views (right) of the white-boxed regions (left) show the degenerating cortical neurons in 30 min BCAL and 60 min BCAL group. **(C)** Quantification of the density of degenerating neurons. The density of FJC positive cells was significantly higher in the 60 min BCAL group (n = 4 mice for each group; ***p*<0.01).

Supplementary Figure 3
**(A)** White-boxed region shows the ROI (region of interest, 0.5×0.5 mm^2^) used to quantify the density of cortical microglia. ROIs were chosen in the area of somatosensory cortex. **(B, C)** The density of cortical microglia at 3 h after BCAL. The microglial density showed no significant difference between three groups (n = 6 mice for each group).

Supplementary Figure 4 Polarization of cortical microglia after ischemia. **(A)** Representative images showing M1 microglia (GFP^+^CD16/32^+^) at 2 days after ischemia in 30 min BCAL group and 60 min BCAL group. White arrows indicate GFP^+^CD16/32^+^ cells. **(B)** Representative images showing M2 microglia (GFP^+^CD206^+^) at 2 days after ischemia in 30 min BCAL group and 60 min BCAL group. White arrows indicate GFP^+^CD206^+^ cells. (**C**) Statistics of the density of M1/M2 microglia in cortex (n = 4 mice for each group; ***p*<0.01, ##*p*<0.01).

Supplementary Figure 5 Differential expressed genes in 60 min BCAL in comparison with 30 min BCAL group. **(A)** Volcano plot shows the upregulation and downregulation of genes in 60 min BCAL group compared to 30 min BCAL group. **(B)** Heatmap showing the differentially expressed microglial markers in different groups. Genes with similar expression pattern were clustered. **(C)** Heatmap showing the differentially expressed endothelial and pericyte markers in different groups. Colors show the relative up- and downregulation of each gene.z

Supplementary Figure 6 Signaling pathways from KEGG database. **(A)** TNF signaling pathway. **(B)** PI3K-Akt signaling pathway. The red box shows differentially expressed genes in 60 min BCAL.
