# Supplementary figures and images for: Differential Regulation of Microglial Activation in Response to Different Degree of Ischemia
Source: Front Immunol. 2022 Jan 28;13:792638. doi: 10.3389/fimmu.2022.792638 (PMC8831277; doi:10.3389/fimmu.2022.792638)

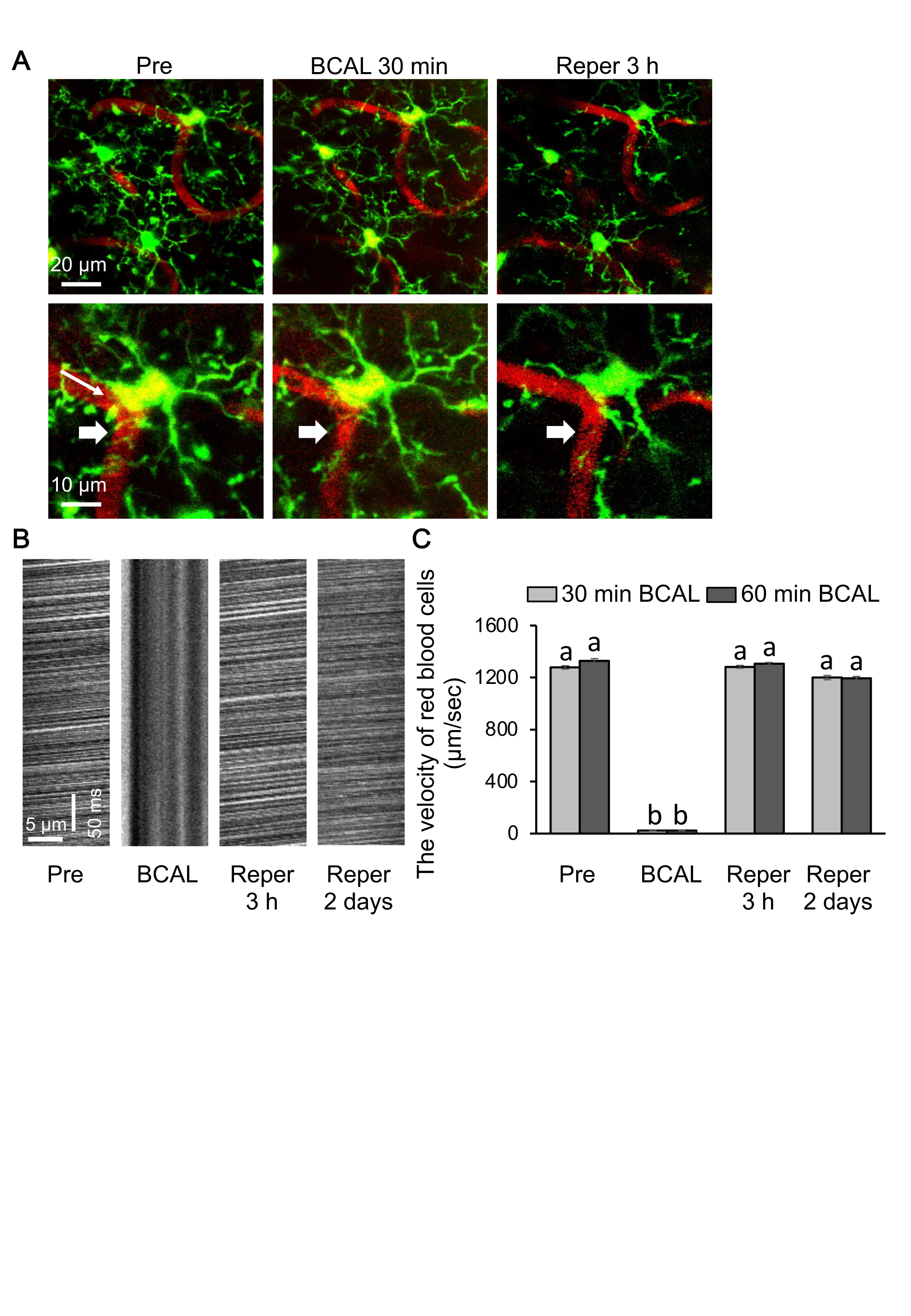

Supplement: Supplementary Figure 1 — Velocity of blood flow. (A–C) Cerebral blood flow before, during and after BCAL were shown as the velocity of red blood cells, respectively. Two-photon images show microglia (green) and vessels (red). Arrows indicate the vessels used for the measurement of blood flow velocity (n = 40 vessels from 5 mice for each group; a vs b, p<0.01). [file Image_1.tif]

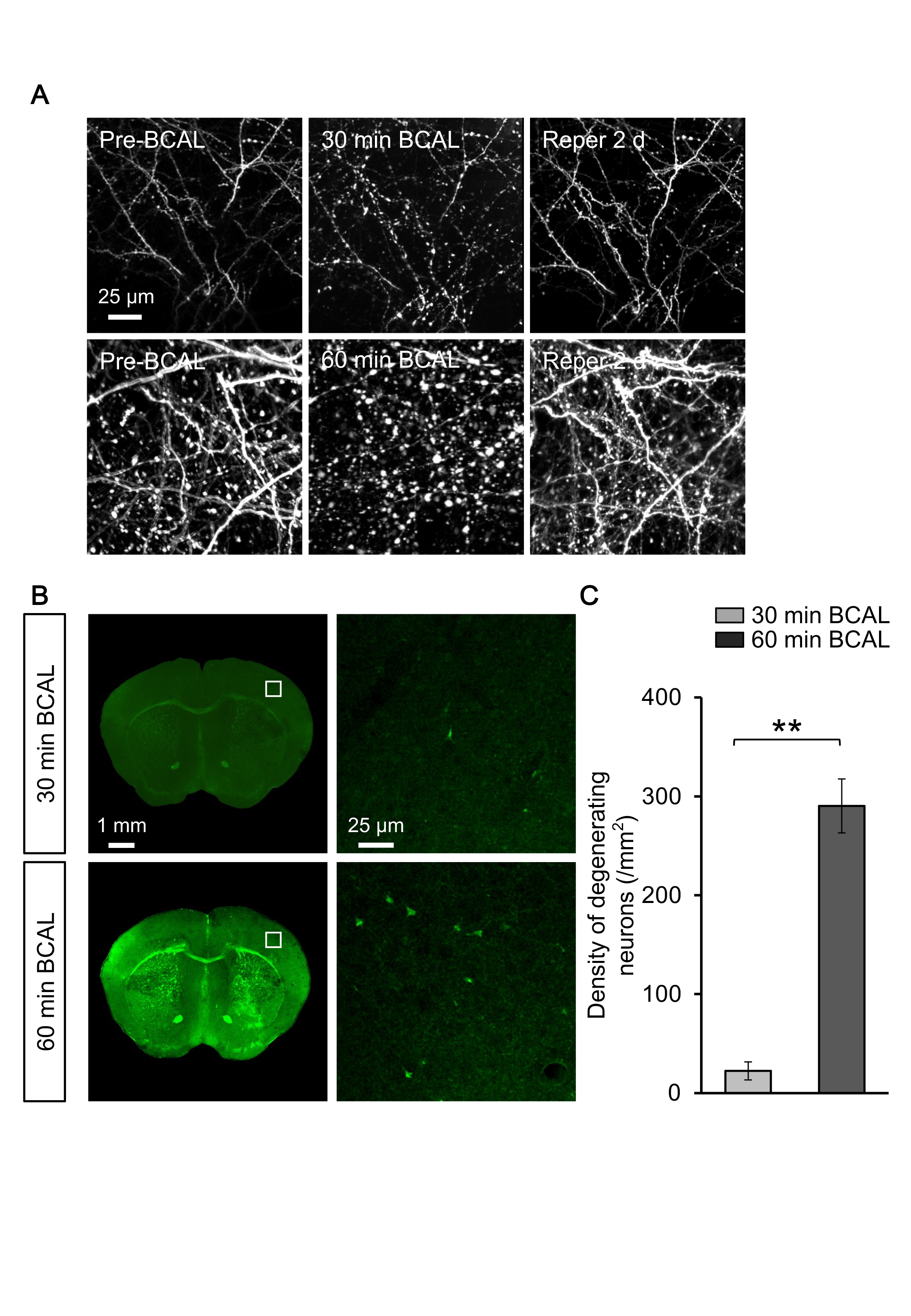

Supplement: Supplementary Figure 2 — Neuronal damage after different degree of ischemia. (A) Beaded-like structural damage of neuronal dendrites induced by 30 min and 60 min BCAL. (B) Magnified views (right) of the white-boxed regions (left) show the degenerating cortical neurons in 30 min BCAL and 60 min BCAL group. (C) Quantification of the density of degenerating neurons. The density of FJC positive cells was significantly higher in the 60 min BCAL group (n = 4 mice for each group; **p<0.01). [file Image_2.tif]

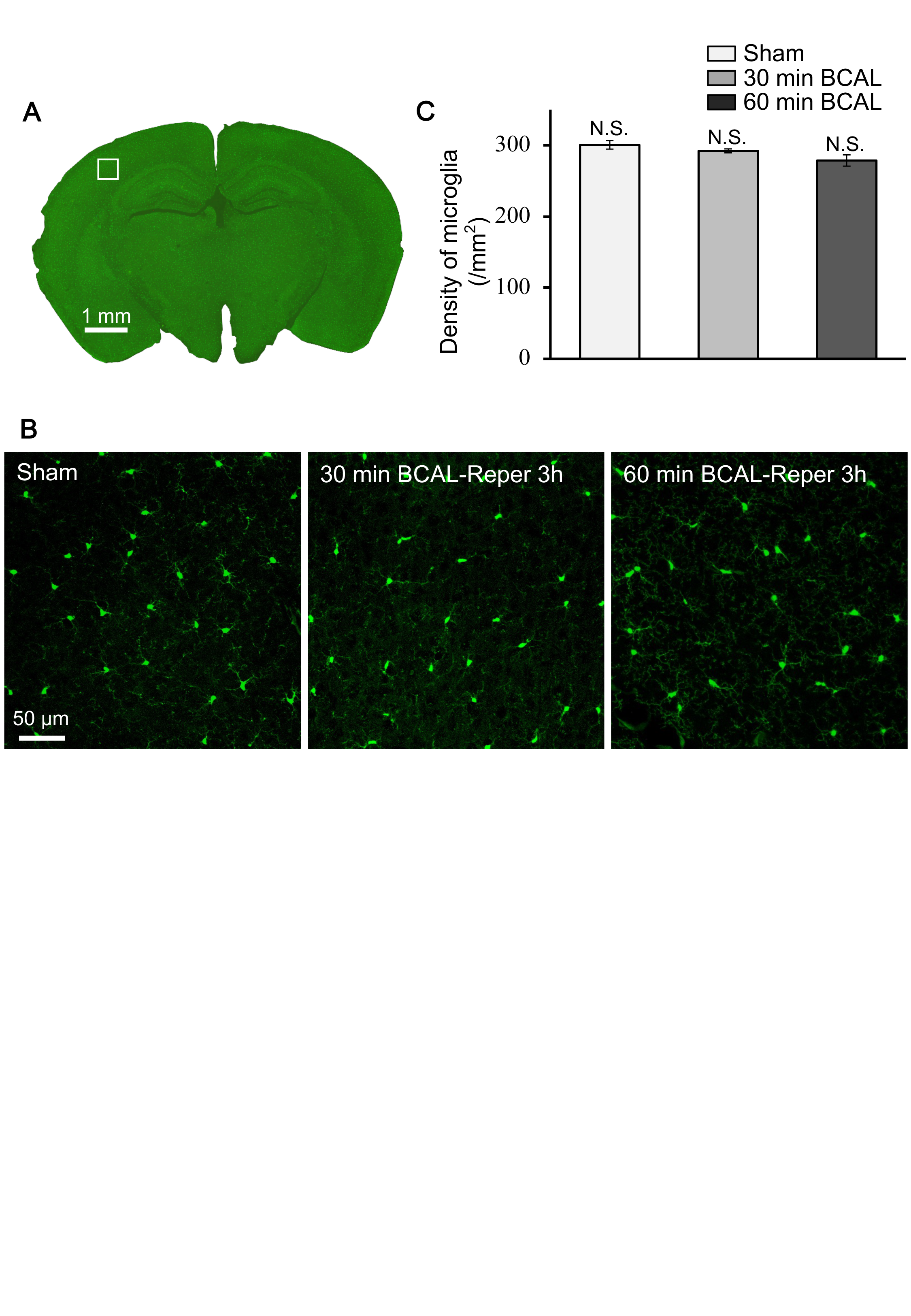

Supplement: Supplementary Figure 3 — (A) White-boxed region shows the ROI (region of interest, 0.5×0.5 mm2) used to quantify the density of cortical microglia. ROIs were chosen in the area of somatosensory cortex. (B, C) The density of cortical microglia at 3 h after BCAL. The microglial density showed no significant difference between three groups (n = 6 mice for each group). [file Image_3.tif]

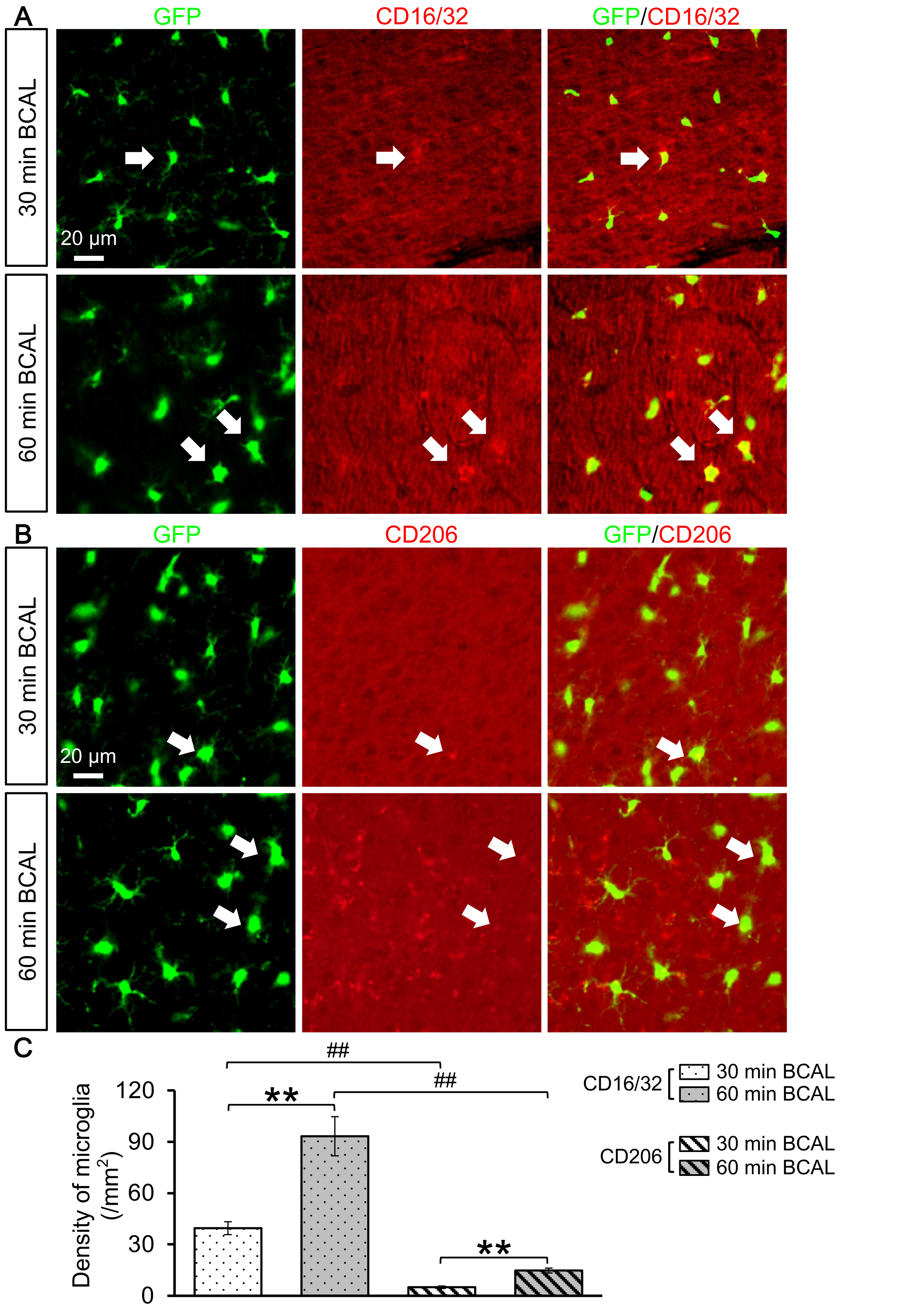

Supplement: Supplementary Figure 4 — Polarization of cortical microglia after ischemia. (A) Representative images showing M1 microglia (GFP+CD16/32+) at 2 days after ischemia in 30 min BCAL group and 60 min BCAL group. White arrows indicate GFP+CD16/32+ cells. (B) Representative images showing M2 microglia (GFP+CD206+) at 2 days after ischemia in 30 min BCAL group and 60 min BCAL group. White arrows indicate GFP+CD206+ cells. (C) Statistics of the density of M1/M2 microglia in cortex (n = 4 mice for each group; **p<0.01, ##p<0.01). [file Image_4.tif]

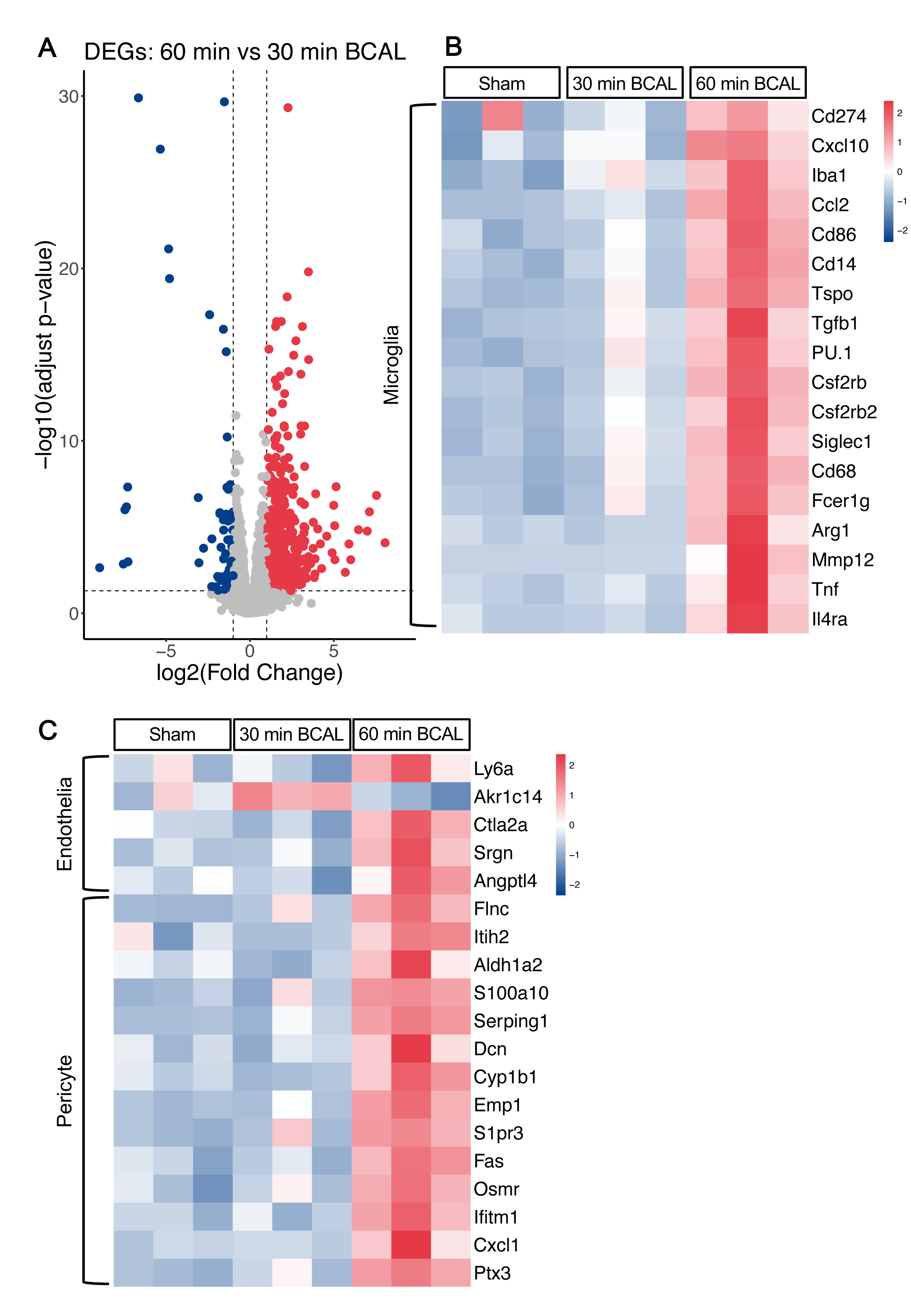

Supplement: Supplementary Figure 5 — Differential expressed genes in 60 min BCAL in comparison with 30 min BCAL group. (A) Volcano plot shows the upregulation and downregulation of genes in 60 min BCAL group compared to 30 min BCAL group. (B) Heatmap showing the differentially expressed microglial markers in different groups. Genes with similar expression pattern were clustered. (C) Heatmap showing the differentially expressed endothelial and pericyte markers in different groups. Colors show the relative up- and downregulation of each gene.z [file Image_5.tif]

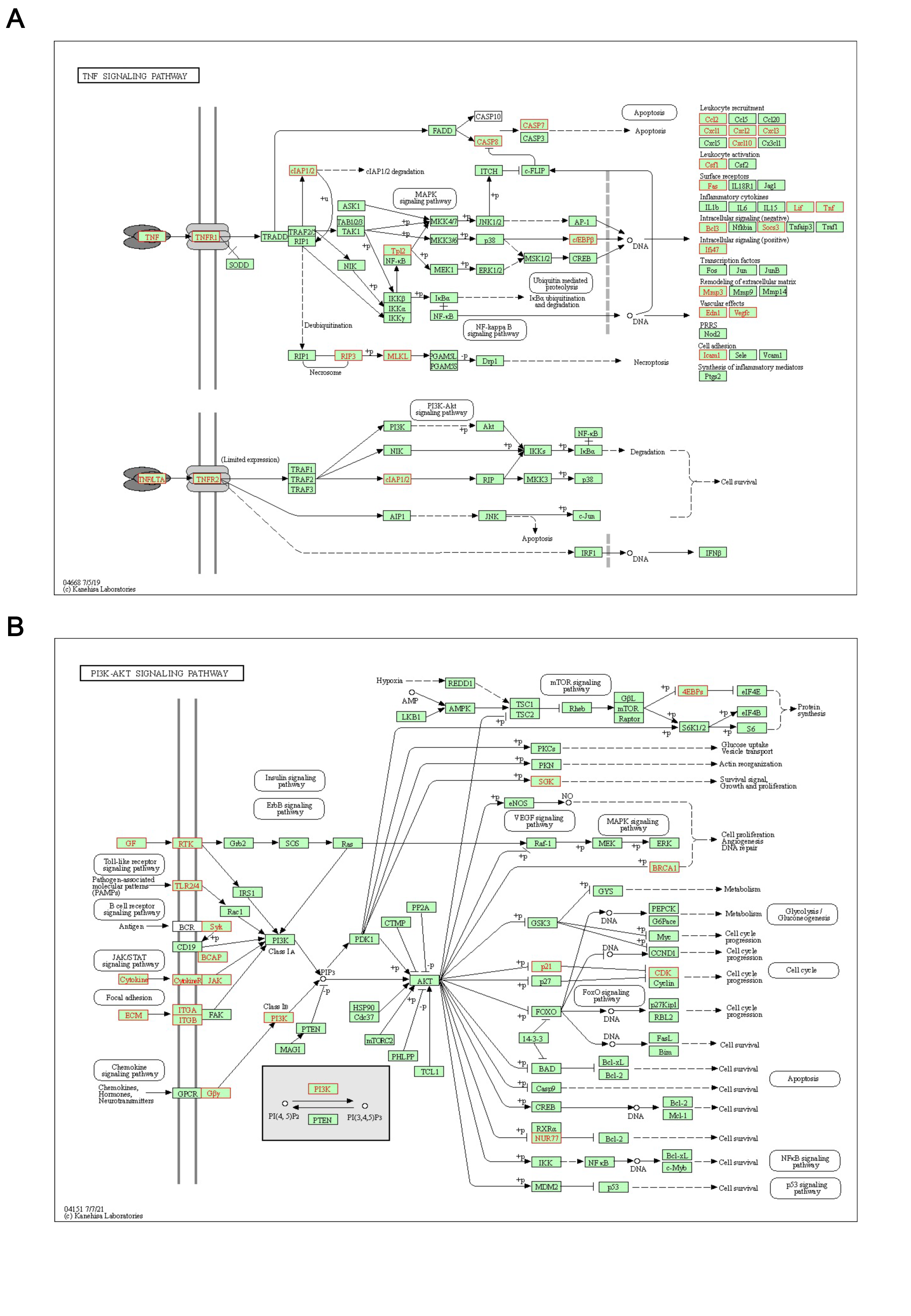

Supplement: Supplementary Figure 6 — Signaling pathways from KEGG database. (A) TNF signaling pathway. (B) PI3K-Akt signaling pathway. The red box shows differentially expressed genes in 60 min BCAL. [file Image_6.tif]
