# Supplementary material for: Differential Regulation of Microglial Activation in Response to Different Degree of Ischemia
Source: Front Immunol. 2022 Jan 28;13:792638. doi: 10.3389/fimmu.2022.792638 (PMC8831277; doi:10.3389/fimmu.2022.792638)
Supplement: Supplementary file 7 [file DataSheet_1.docx]

## Supplementary Methods

**Immunofluorescence staining**

To label M1 and M2 polarized microglia, we performed CD16/32 and CD206 staining. Briefly, the brain sections (30 μm) were permeabilized with 0.1% Triton X-100 for 10 min and blocked with 5% BSA for 1 h. Then the sections were incubated with primary antibodies (CD16/32, 1:50, BD, CAT# 553142; CD206, 1:100, R&D, CAT# AF2535) overnight at 4 ℃ followed by the incubation with respective secondary antibodies (rhodamine-conjugated goat anti-rat, 1:100, Proteintech, CAT# SA00007-7; rhodamine-conjugated donkey anti-goat, 1:100, Proteintech, CAT# SA00007-3) for 1 h at room temperature. Images were acquired by using an Olympus microscope. The cellular density was quantified by using ImageJ software.

**Fluoro-Jade C (FJC) staining**

To assess the damage to cortical tissue caused by different degrees of ischemia, we used Fluoro-Jade C staining to label degenerating neurons (Schmued et al., 2005). Briefly, brain sections (30 μm) were mounted on gelled slides and dried in 37 ℃ overnight. The slides were then transferred into 0.06% potassium permanganate solution and incubated for 10 min (protected from light). After being rinsed in distilled water, the slides were immersed in 0.0001% FJC staining solution (prepared with 10% acetic acid) for 10 min. Then the slides were rinsed, dehydrated by 100% ethanol, transparentized with and stored with DPX (Sigma). All slides were then examined by an Olympus epifluorescence microscope and an Olympus confocal microscope.

**References**

Schmued, L. C., Stowers, C. C., Scallet, A. C., and Xu, L. (2005). Fluoro-Jade C results in ultra high resolution and contrast labeling of degenerating neurons. *Brain Res.* 1035, 24–31. doi:10.1016/j.brainres.2004.11.054.
